# The Employee Relationship Analysis on Innovation Behavior of New Ventures Under the Organizational Psychology and Culture

**DOI:** 10.3389/fpsyg.2022.804316

**Published:** 2022-03-09

**Authors:** Sijin Du, Jianjun Wang

**Affiliations:** College of Finance and Economics, Qinghai University, Xining, China

**Keywords:** human resource management system, organizational psychological ownership, sense of organizational support, transformational leadership, psychology, behavior

## Abstract

The study aims to explore the psychology and behavior of employees in organizations in enterprise innovation. Based on the human resource management system (HRMS), organizational psychological ownership, and other related theories, the transformational leaders and their advice behavior in start-ups are taken as the research object. The data obtained from the questionnaire as the research samples. Second, the influence and intermediary effect of employees’ organizational psychological ownership on colleagues, leaders, and the whole enterprise are discussed, and the corresponding conclusions are drawn. The results show that the path coefficients of transformational leaders of start-up enterprises for employees’ advice to their superiors and their peers are 0.28 and 0.31, respectively, and *p* < 0.01. Therefore, transformational leadership has a positive impact on both elements. In the relationship between organizational psychological ownership and employee creativity, the *r* value is 0.34 and *p* < 0.01. This shows that organizational psychological ownership positively correlates with employees’ creativity. In addition, corporate support can mediate employees’ behavior and psychological ownership in the organization and has a positive correlation in support, identity, and care. Therefore, the impact of organization-employees relations on employees’ innovation behavior is discussed based on organizational psychology and culture, which can improve employees’ subjective initiative for work and provides ideas for the management and development of start-ups.

## Introduction

With the increasing competition in the market, an enterprise needs to improve itself from all aspects and make itself more flexible, innovative, and adaptable. This requires the enterprise members to play their subjective initiative and actively express their opinions and suggestions (Cheng et al., 2021). Employees’ advice can promote innovative thinking and improve their ability to resist threats from other enterprises. Employees’ advice includes their critical attitudes, the help to realize the enterprise’s goal, and constructive behavior, which are not their tasks (Holscher et al., 2020). Enterprises can enable employees to transform their job dissatisfaction into innovative thinking and improve their performance through expressing their opinions openly. In the enterprise, the leader should assign employees and encourage them to do more out of their obligation (Salehi et al., 2020). In general, leaders’ credibility will have a particular impact on employees’ psychological safety index, which will affect employees’ voice behavior. Transformational leadership is different from traditional leadership. Instead of motivating employees only through rewards and punishments, transformative leadership focuses on the value and significance of the work and organization, pays attention to the responsibility and commitment, and shifts employees’ focus from personal interests to collective interests (Wilaj, 2019; Darwish, 2020; Baluku et al., 2021). In the theory of self-regulation orientation, transformational leadership is positive and risk-taking. Employees’ voice behavior can be divided into higher-level progressive advice and friendly, progressive advice. At the same time, it contains three characteristics of extra-role, challenge status, and risk response.

As a driving force, Human Resource Management System (HRMS) can help enterprises improve their competitiveness in the market and has received much attention (Qi and Yao, 2021). Many researchers argue that HRMS is related to technology, knowledge, and products and has a considerable impact on the operation of enterprises. There are many different views on HRMS, which are mainly divided into two types. One is that HRMS is conducive to improving enterprise innovation (Kim, 2019). In other words, HRMS favors flexible design in the innovation and management of products. The other is that HRMS harms enterprise innovation. American scholars Bastan et al. (2020) collected relevant data from 200 enterprises and analyzed employees’ organizational psychological ownership and innovation ability. The results show that most HRMS practice variables positively correlate with enterprises’ innovation ability, while performance has a negative correlation with employees’ organizational psychology. Therefore, performance cannot improve enterprises’ innovation ability (Bastan et al., 2020).

For employees in the enterprise, perceived corporate support positively impacts employees’ attitudes and even behavior. Therefore, psychological variables are gradually introduced into the organization, forming the concept of organizational psychology (Qian et al., 2018), and psychological capital, psychological empowerment, and psychological ownership play a role in promoting organizational psychology. Corporate psychological ownership is the manifestation of employees’ subjective initiative awareness. If employees regard the enterprise as theirs, they will devote themselves to its development (Rados et al., 2018; Chen, 2019). Research on employees’ organizational psychology can better predict employees’ attitudes toward enterprises and their tasks. Perceived corporate support has a particular impact on psychological ownership and behavior. Yadav et al. (2020) discussed improving the relations between employees from psychology, sociology, and management. Through the theoretical research, improving personnel quality can be summarized as: First, improving the relations between employees reflects an organizational atmosphere, and it is an attribute that can be measured. Second, it has an indelible impact on corporate production. Third, it is the perception of organizational members about their organizational environment. This perception comes from the work experience of members. It also affects members’ motivation, attitudes, beliefs, values, and behavior. Finally, empirical research is conducted to explore employees’ relations (Yadav et al., 2020). Based on the social exchange theory, Wang et al. (2019) examined the relationship between employment security, organizational support, and enterprise innovation and analyzed the role of employment security in enterprise innovation through instrumental and emotional paths according to “organizational practice – employees’ psychological perception – employees’ behavior” (Wang et al., 2019). Zhang et al. (2019) studied the expectation of leaders to subordinate employees, the horizontal exchange between employees, and the impact of transformational leadership on innovation. They tried to implement a theoretical model that has a mediating effect and regulatory effect of different leadership styles and employees’ relations on enterprise innovation (Zhang et al., 2019). Garidis and Rossmann (2019) found that the evaluation-oriented performance appraisal was negatively correlated with the enterprise’s innovation. The development-oriented performance appraisal was positively correlated with the enterprise’s creativity through the survey of 389 employees. The organizational innovation atmosphere plays a partial intermediary role in the impact of the performance appraisal on the enterprise’s innovation (Garidis and Rossmann, 2019).

**Figure 1:**
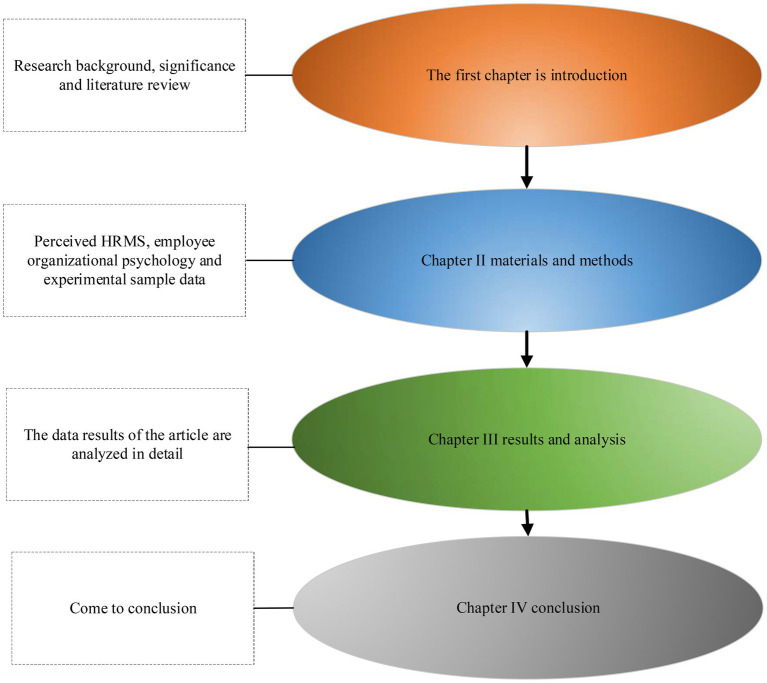
Organizational structure of the article.

From the perspective of organizational psychological ownership and innovation behavior of start-ups, the impact of the four-quadrant types in HRMS on employees’ creativity is expounded. The effects and functions of employees’ organizational psychological ownership on colleagues, leaders, and the whole enterprise are discussed. According to the relevant theoretical system, the experts and scholars explore employees’ relations at the level of organizational psychology and culture from different angles. Compared with the theories or methods proposed by the above experts and scholars, the impact of employee relations on the innovation behavior of start-ups is systematically analyzed. The study provides a reference for the research on the effect of employee relations based on organizational psychology and culture on the innovation behavior of start-ups. And the specific organizational structure is shown in Figure 1.

## Perceived HRMS and Employees’ Organizational Psychology Under New Enterprise Leadership

### Perceived Differences in HRMS

#### Definition of HRMS

**Figure 2:**
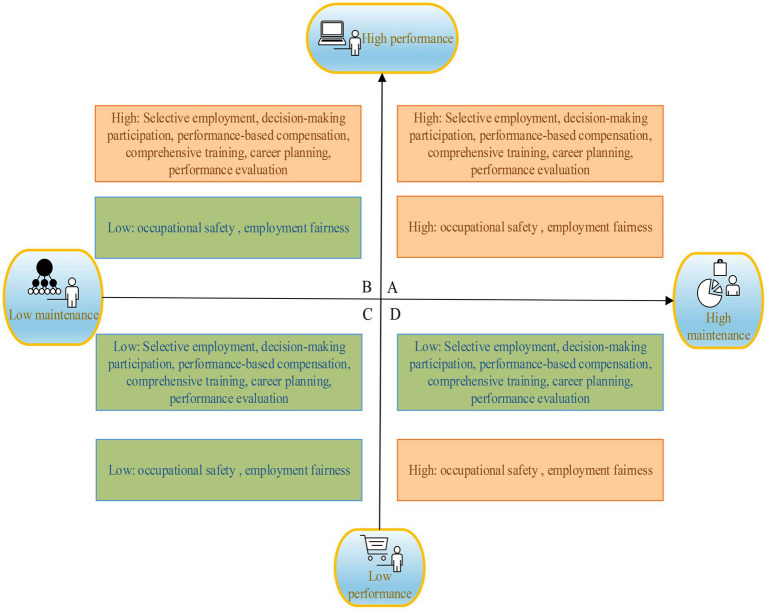
Four classes of HRMS.

The human resource management system is called HRMS. According to practice content, HRMS can be divided into several departments with different functions (Mcclean and Collins, 2019). In HRMS, performance and maintenance play different roles. For maintenance-oriented HRMS, there is usually a focus on employees’ stability and fairness, which are not related to the value of the enterprise’s production ratio. Performance-oriented HRMS often pays more attention to completing the tasks and the enterprise’s profits, and its goal is to improve productivity. At the same time, these two different types of HRMS also have different needs for employees. Performance-oriented HRMS mainly enriches employees’ knowledge and improves their skills through training to make them meet the needs of enterprises, which maintenance-oriented HRMS focuses on the satisfaction and security of employees (Saks, 2021). In HRMS, performance orientation and maintenance orientation are two independent modules, and they need to be analyzed from different dimensions. Here, the four-part diagram method in management is used to classify the two modules and combine them, and four types of HRMS are obtained. According to the different degrees of practice, as shown in Figure 2, employees’ perceptions are divided into four classes. Class A is defined as “high performance and high maintenance,” class B as “low performance and high maintenance,” class C as “high performance and low maintenance,” and class D as “low performance and low maintenance.”

- For the employees with the perception of high maintenance and performance, organizations can maintain employees’ sense of security by commitment and commitment and improve employees’ work efficiency and create benefits for enterprises through performance incentives. Such HRMS can maintain a stable long-term relationship between enterprises and employees.
- For the employees with low performance and high maintenance, organizations should give employees a sense of security instead of work plans and assessments. Such systems can still maintain a stable relationship between enterprises and employees, while commitment to employees can also promote job performance.
- For the employees with the perception of high-performance and low-maintenance, employees in the enterprise are subject to high-performance requirements and a lack of security. It is not easy to maintain a long-term relationship between enterprises and employees. However, if there are clear task plans and requirements, employees and enterprises can maintain a short-term relationship.
- For the employees with the perception of low performance and low maintenance, HRMS is difficult to play its role because employees’ performance and job security have not been paid attention to and guaranteed. Such a relationship between enterprises and employees cannot continue.

#### Differences of HRMS

In the performance dimension of HRMS, performance-oriented training for employees and encouraging employees to participate in management can help employees acquire knowledge and skills and find ways to improve themselves, increasing employees’ competency to the position. Career planning and performance assessment make employees know about their development direction and enterprises’ current situation, which gives employees a sense of belonging to enterprises.

In the maintenance dimension of HRMS, HRMS focuses on providing employees with employment security so that employees are willing to spend energy and time acquiring knowledge and skills. In this way, organizations become places where employees can express themselves and release their emotions. Employees and enterprises are not independent of each other but belong to each other. They think of themselves as part of the enterprise and have a sense of belonging, thereby enhancing the psychological ownership of the organization. According to the individual and organization matching theory, if the human resource management meets the various needs of employees, it will play a synergistic role. Performance-oriented human resource management can help acquire employees’ skills and promote their career development. Maintenance-oriented human resource management can give employees a sense of security and belonging. The more needs of employees that the human resource system meets, the more positive attitudes toward their tasks employees will have. Therefore, compared with the other three types of human resources systems, the HRMS with high performance and high maintenance has the most significant positive impact on employees’ organizational psychological ownership. In contrast, low performance and low maintenance have the most negligible impact on employee corporate psychological ownership.

Employees have high-level needs such as skill acquirement and career development and have low-level needs such as work safety and fairness. The satisfaction of low-level needs can promote the production and fulfillment of high-level requirements. High maintenance-oriented human resource management can enhance the effect of performance-oriented human resource management on employees’ organizational psychological ownership. Low maintenance-oriented human resource management can reduce performance-oriented human resource management’s impact on employees’ corporate psychological requests. Based on the above, the following hypotheses are proposed as:

*a1:*In class A, the organizational psychological ownership is highest.

*a2:*In class B, the corporate psychological ownership is more heightened.

*a3:*In class C, the managerial psychological ownership is lower.

*a4:*In class D, the organizational psychological request is most insufficient.

Usually, individual innovation is complex and uncertain. When employees have organizational psychological ownership of the enterprise, they view themselves as its owners. They can take the initiative to think about effective ways to achieve long-term development of the enterprise, be concerned about the organization’s long-term interests in decision making, and even urge other employees to take the initiative to take organizational risks for the organization. Corporate psychological ownership focuses on employees’ performance in the organization. When employees’ psychological investment is high, they will have a sense of responsibility for the organization. They are devoted to solving the problems encountered by careful thinking and comprehensive reconstruction. Finally, the corresponding solutions are figured out. When his psychological input is higher, the employee’s dependence on organizational identification and emotion is more substantial, conducive to stimulating his creativity and organizational vitality. Therefore, the hypothesis is proposed as:

*a5:*Organizational psychological ownership has a positive impact on employees’ creativity.

### Transformational Leadership and Admonition

Transformational leadership stimulates employees’ needs by making employees realize the significance of the tasks they undertake and actively establishing an atmosphere of mutual trust to encourage employees to sacrifice their interests for the interests of the enterprise. It can be divided into four parts: leadership charm, vision encouragement, intelligent inspiration, and personalized care. In the domestic cultural background, transformational leadership generally includes joy, vision encouragement, customized care, and a virtue model. Vision encouragement requires leaders to explain the prospect and significance of the enterprise to employees and clarify their development direction. Leadership charm refers to the leader’s ability and innovative ideas. In domestic transformational leadership, the connotation of personalized care is more extensive. It pays attention to employees’ performance and development and their family and public relations. The virtue model means that leaders should have the spirit of dedication and sacrifice, and their behavior should set an example to employees and match their words and deeds (Wu and Song, 2019).

Leaders with transformational leadership can create more attractive visions for their employees, give employees personalized care and encourage, and accept innovative thinking. Such leadership can arouse employees’ enthusiasm for work and their extra-role behavior.

Giving advice is also a kind of extra-role behavior, and it requires employees to make extra efforts and even the need to bear certain risks. Transformational leadership can make employees know the overall goal of the enterprise by shaping the vision and behaving appropriately, and striving for the goal instead of personal interests. According to the posts, advising behaviors can generally be divided into two types: advice to supervisors and colleagues. Being familiar with each other’s work content and situation helps to find problems in time and get solutions, improving their work efficiency. Although advising behaviors are extra-role, challenging, and risk-taking, transformational leadership is helpful to supervisors and colleagues. Therefore, the following hypotheses are proposed as:

*c1:*Transformational leadership positively impacts employees’ advice to supervisors.

*c2:*Transformational leadership positively impacts employees’ direction to colleagues.

### Mediating Effect of Organizational Psychological Ownership

Psychological ownership is a mental state that produces a sense of ownership of the target based on possession psychology. In an organization, when members have a sense of ownership of the organization, their psychological states become the psychological ownership of the organization. Usually, the elements of psychological ownership include the control target, intimate understanding, and individual input (Pellerone et al., 2019). Organizational psychological ownership can meet the three needs of human beings for a sense of belonging, self-identity, and self-efficacy. When people control something, they will eventually lead to psychological feelings of ownership. When there are more control behaviors, things will gradually be considered their own (Wu et al., 2018).

High performance-oriented human resource management can better arouse employees’ creativity than low performance-oriented human resource management by influencing employees’ organizational psychological ownership. Under the same performance-oriented human resource management, high maintenance-oriented human resource management helps enhance employees’ job stability so that employees feel that their work in the enterprise is not short-term but stable (Wu et al., 2017, 2020; Yuan and Wu, 2020).

Compared with low maintenance-oriented human resource management, high maintenance-oriented human resource management can improve employees’ psychological ownership and creativity. Based on the above discussion, the following hypotheses are proposed as:

*d1:*Organizational psychological ownership plays a mediating role between the Class A, Class B, Class C, and Class D of HRMS and employees’ creativity.

*d2:*Compared with other classes, organizational psychological ownership has the most substantial mediating effect between Class A of HRMS and employees’ creativity. Thus the employee creativity of Class A of HRMS is the highest.

Also, corporate psychological ownership contains the concept of “home,” which makes employees feel safe in the organization, and the risk of being blamed because of giving advice is eliminated. Since organizational psychological ownership allows employees to view the organization as part of their own, employees will produce the idea and cognition of “this organization is mine” when giving advice (Zheng et al., 2018). Admonition can improve the efficiency and competitiveness of the organization and ultimately make employees achieve their benefits. Therefore, organizational psychological ownership can arouse employees’ working enthusiasm. Transformational leadership affects employees’ corporate psychological rights, affecting their voice behavior. Thus, the following hypotheses are proposed as:

*d3:*The mediating effect of organizational psychological ownership between transformational leadership and employees’ advice to supervisors.

*d4:*Organizational psychological ownership plays a mediating role in advising colleagues.

### Sample Data Integration

A total of 10 enterprises in City J and City X are selected, and 500 questionnaires are distributed online. A total of 462 valid questionnaires are recovered, with a recovery rate of 92.4%. The questionnaire is divided into two parts. The first part is for employees, and the other is for leaders. The questionnaires are distributed to 400 employees, and 377 valid questionnaires are recovered, with a recovery rate of 94.2%. Hundred questionnaires are distributed to leaders, and 85 are recovered, with a recovery rate of 85%.

The questionnaire results are analyzed by the 7-point Likert Scale and 5-point Likert Scale. The performance-oriented HRMS and maintenance-oriented HRMS are analyzed using the 7-point Likert Scale, and employee creativity is analyzed using the 5-point Likert Scale (Oosterveld et al., 2019). The performance-oriented questionnaire includes six questions, namely, employment selection, decision-making participation, salary performance, training extension, career planning, and performance evaluation, with 36 queries. A maintenance-oriented questionnaire includes employment security and fairness with 12 questions. Performance-oriented and maintenance-oriented HRMS are taken as dimensions. The HRMS is divided into four classes by K-means. The dummy variable of HRMS is constructed to explore the influence of four courses of HRMS on organizational psychological ownership and employees’ creativity (Li et al., 2019). A total of seven questions are set to measure the psychological request of individuals and shared organizations, and 13 questions are put to measure their creativity. For transformational leadership and voice behavior, a total of 26 questions are designed, including eight for moral models, six for vision encouragement, six for leaders’ charm, and six for personalized care, and the 5-point Likert Scale scores the 26 questions. The voice behavior scale includes nine questions to supervisors and six to colleagues and 6-point Likert Scale scores for the 15 questions.

The aggregate validity and discriminant validity are tested by confirmatory factor analysis (CFA). Based on LISERAL 8.7, confirmatory factor analysis is conducted on performance-oriented human resource management, maintenance-oriented human resource management, employee creativity, transformational leadership, advising supervisors and colleagues, and organizational psychological ownership. The mediating effect is tested according to four conditions (Ecuyer et al., 2019): (a) independent variables have a significant influence on dependent variables; (b) Independent variables have substantial impacts on mediating variables; (c) The mediating variables have substantial effects on the dependent variables; and (d) When the effect of mediating variables is controlled, the impact of independent variables on dependent variables is not significantly weakened.

## Data Inspection and Result Analysis

### Reliability and Validity Test of Scale

SPSS is used to analyze the reliability and validity of 562 valid sample data obtained from the questionnaire. It is necessary to test the reliability and validity of the measurement scale to ensure the credibility of statistical conclusions. The *KMO* coefficient is introduced to verify them. The specific calculation method is shown in equation (1).


(1) $$KMO=\frac{\sum \sum_{i\neq j}r_{ij}^{2}}{\sum \sum_{i\neq j}r_{ij}^{2}+\sum_{i\neq j}r_{ij•1,2\ldots k}^{2}}$$

In equation (1), r represents the reliability coefficient, i represents the dependent variable, j represents the independent variable, and k represents the quantity. The specific measurement standards of KMO are shown in Table 1.

**Table 1:** Measurement standard of *KMO*.

Type	Range of values	Factor analysis
KMO	<0.9	Appropriate
	0.8 ~ 0.9	Extremely appropriate
	0.7 ~ 0.8	Appropriate
	0.6 ~ 0.7	Not appropriate
	0.5 ~ 0.6	Almost appropriate
	>0.5	Appropriate

Based on statistical knowledge, the effectiveness of the designed questionnaire data is analyzed. The value of KMO is 0.869, ranging from 0.8 to 0.9, and the *p* value is 0, less than 0.01. Therefore, these data are very suitable for factor analysis, and the questionnaire has good effectiveness.

According to equation (1), the score difference of the actual number can also be obtained, as shown in equation (2).


(2) $$\alpha =\frac{K}{K-1}\left(1-\frac{\sum_{i=1}^{K}\sigma_{j}^{2}}{\sigma_{i}^{2}}\right)$$

In equation (2), *α* is the coefficient, *K* is the quantity, and other letters have the same meaning as the above equations. Generally speaking, the higher the reliability coefficient is, the higher the reliability between variables is, and the higher the internal consistency between variables is. When *α* is less than or equal to 0.3, the variable is not trusted; when it is between 0.3 and 0.4, the variable is initially credible; when it is between 0.4 and 0.5, there is a slight credibility between variables; when it is between 0.5 and 0.7, the variables are trusted; when it is between 0.7 and 0.9, the variables are credible; and when it is greater than 0.9, the variable is very credible. Through equation (2), the reliability of internal consistency of the answers to the questionnaire is tested, and the calculated result is 0.86, indicating that the questionnaire is reliable.

**Figure 3:**
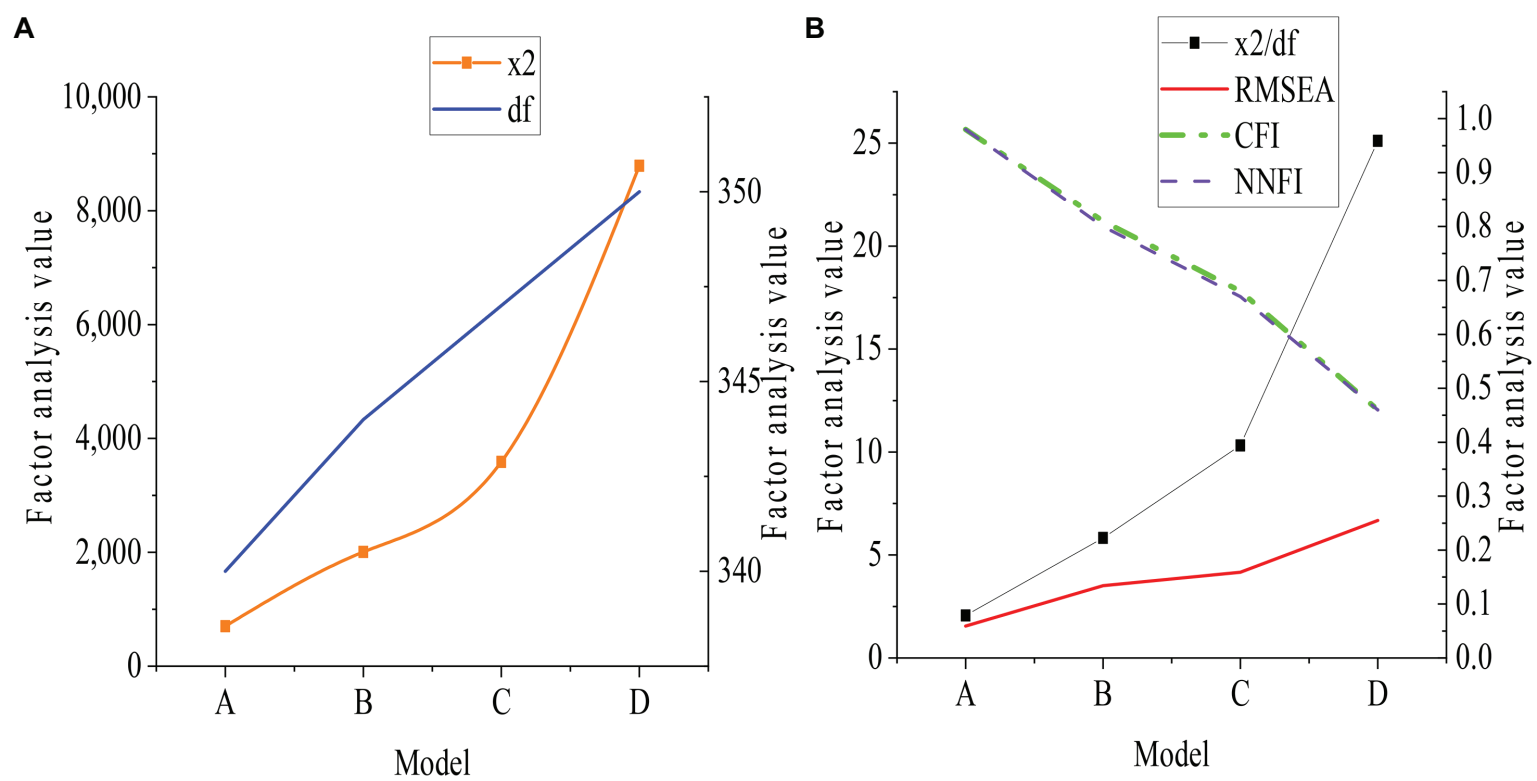
Confirmatory factor analysis results of transformational leadership and advice behavior based on the questionnaire **(A)**: Chi-square and degree of freedom; **(B)**: Chi-square degree of freedom, CFI, RMSEA, and NNFI.

Chi-square χ^2^, Degree Of Freedom (df), Chi-square 2/df, Comparative Fit Index (CFI), Root-mean-square Error Of Approximation (RMSEA), and Non-Normed Fit Index (NNFI) are selected. A is defined as a four-factor model consisting of transformational leadership, organizational psychological ownership, and advising supervisors and colleagues, B as a three-factor model comprised of transformational leadership, corporate psychological ownership, and advising supervisors and colleagues, C as a two-factor model consisting of transformational leadership, organizational psychological ownership, providing guidance to supervisors, and advising colleagues, and D as a single-factor model consisting of transformational leadership, corporate psychological ownership, and advising supervisors and colleagues. The results are shown in Figure 3.

**Figure 4:**
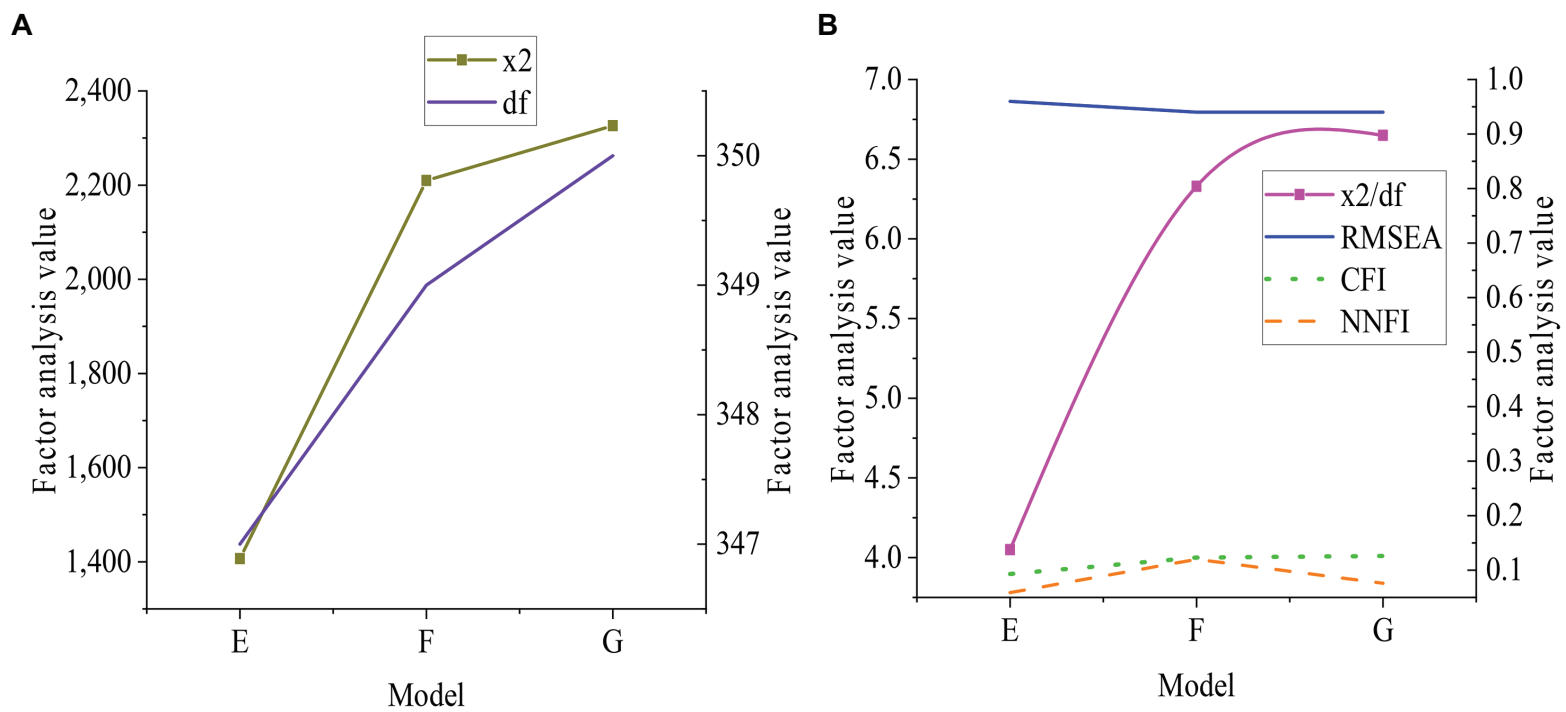
Confirmatory factor analysis of human resources management **(A)**: Chi-square and degrees of freedom; **(B)**: Chi-square degrees of freedom, CFI, RMSEA, and NNFI.

*E* is a three-factor model composed of performance orientation, maintenance orientation, and employee creativity. F is a two-factor model, including performance orientation, maintenance orientation, and employee creativity. G is a single-factor model, and its elements are performance orientation, maintenance orientation, and employee creativity. The results are shown in Figure 4.

Figures 3, 4 show that a load of each index is between 0.5 and 1, and T is between 9.8 and 21.92. And they can test the convergent validity and discriminant validity of each index of the survey. The scale designed has good convergent validity and discriminant validity.

### Hypothesis Verification

**Figure 5:**
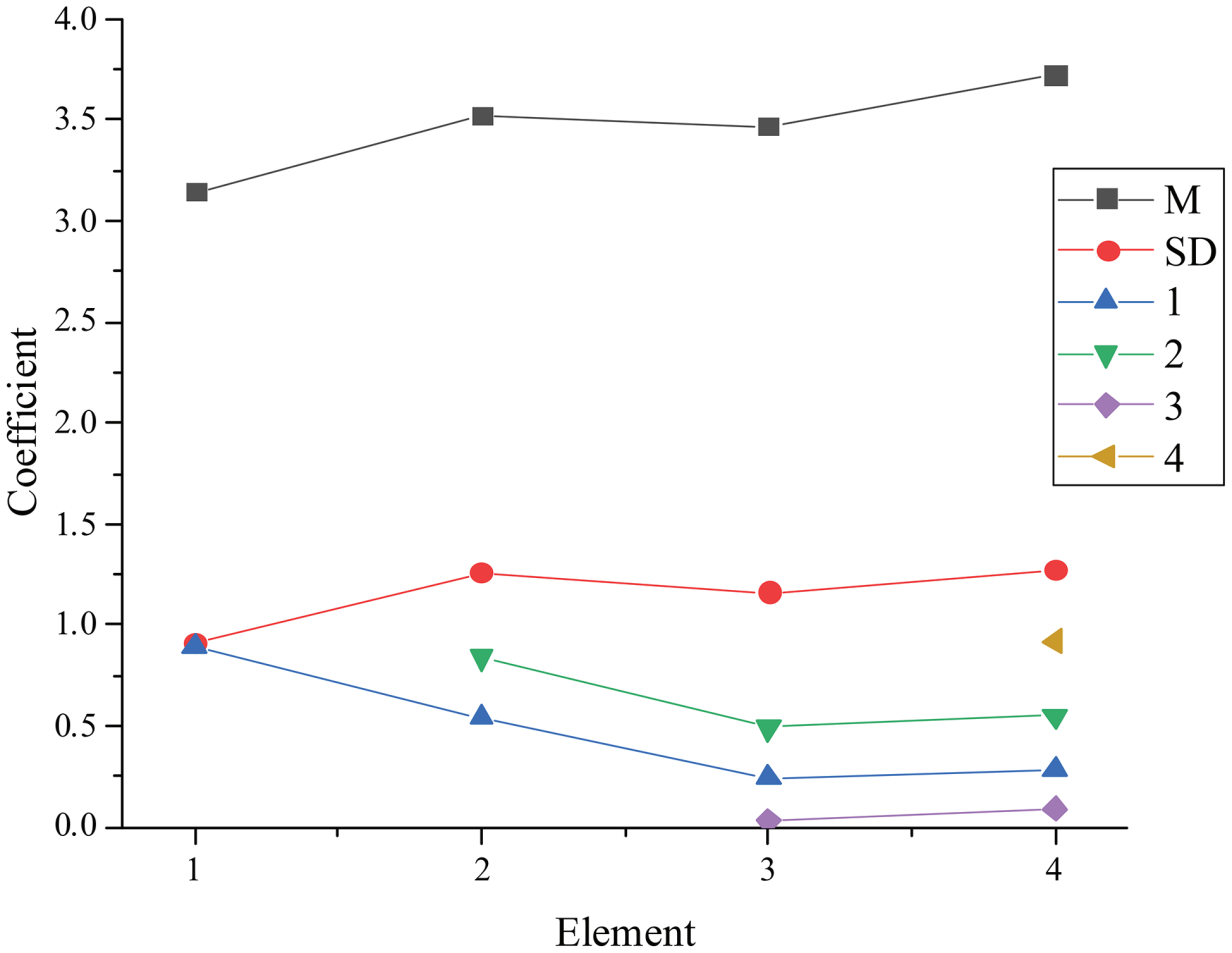
Test results of hypotheses c1 and c2 (*M* is the average value, SD is the standard deviation, 1 is the transformational leadership, 2 is the organizational psychological ownership, 3 is the advice to supervisors, and 4 is the advice to their peers).

The mean (*M*), standard deviation, and path analysis are analyzed. Transformational leadership is defined as 1, organizational psychological ownership as 2, advising supervisors as 3, and colleagues as 4. Hypotheses c1 and c2 are verified, and the coefficients of the test results are shown in Figure 5.

According to the above results, the coefficients of employees’ advice to supervisors and their peers are 0.28 and 0.31, respectively, and *p* < 0.01. Therefore, transformational leadership has a positive impact on both. Hypotheses c1 and c2 are verified, and they are valid.

**Figure 6:**
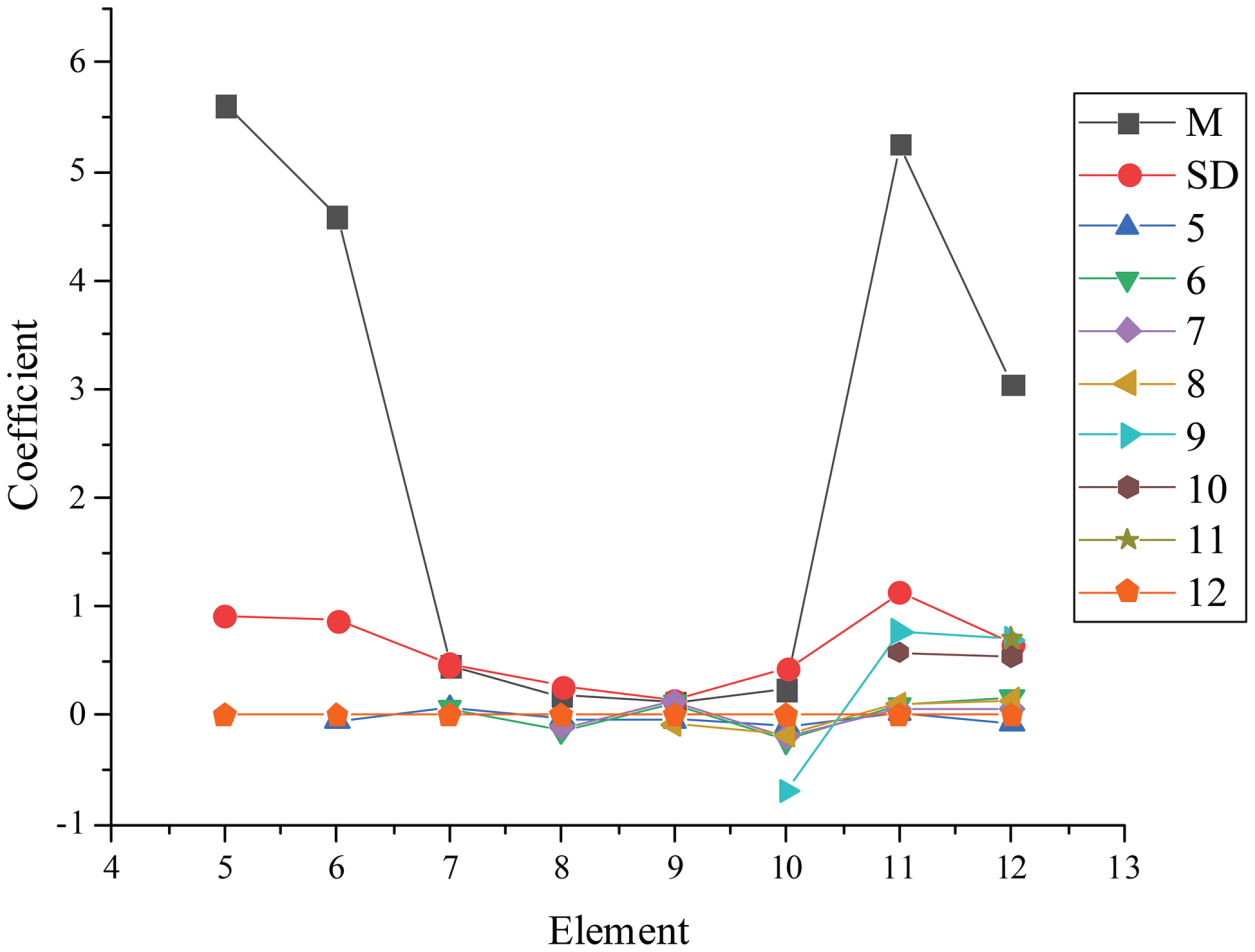
Test results of hypotheses a1 to a5 (*M* is the average value, SD is the standard deviation, 5 is the performance orientation, 6 is the maintenance orientation, 7 is the type of the first quadrant, 8 is the type of the second quadrant, 9 is the type of the third quadrant, 10 is the type of the fourth quadrant, 11 is organizational psychological ownership, and 12 is employees’ creativity).

The research defines the performance orientation as 5, the maintenance orientation as 6, Class A as 7, Class B as 8, Class C as 9, Class D as 10, organizational psychological ownership as 11, and employees’ creativity as 12 to verify hypotheses a1, a2, a3, a4, and a5. The coefficients of the test results of the five hypotheses are shown in Figure 6.

Figure 6 shows that the r value between performance orientation and psychological ownership is 0.76 and *p* < 0.01, indicating that they are positively correlated. The r value between maintenance orientation and psychological ownership is 0.58 and *p* < 1. This suggests that there is a positive correlation between them. In the test of the relationship between four HRMS and psychological ownership, the r value of the first quadrant is 0.68 and *p* < 0.01, proving that their relationship is positive. The *r* value of the second quadrant is −0.08, *p* < 0.11, and the connection is not significant. The *r* value of the third quadrant is −0.11, and its *p* value is less than 0.05, so there is a negative correlation between them. The *r* value of the fourth quadrant is −0.68, and its *p* value is less than 0.01, which indicates a negative relationship between them. In the relation between organizational psychological ownership and employee creativity, the *r* value is 0.34 and *p* < 0.01. Therefore, organizational psychological ownership has a positive correlation with employee creativity. Hypotheses a1, a2, a3, a4, and a5 are verified.

### Test for the Mediating Effect

**Figure 7:**
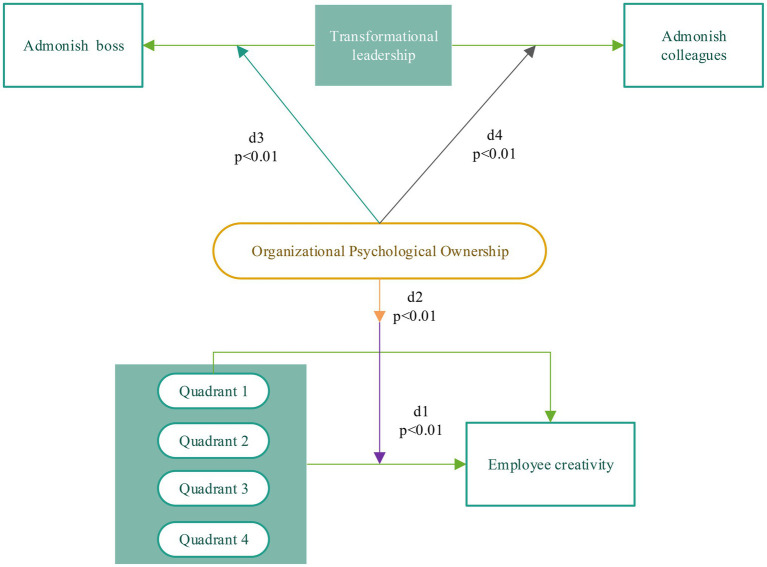
Mediating effect modelling and *p* value test.

The relationship is modeled and the *p* values of d1, d2, d3, and d4 are obtained, as shown in Figure 7.

**Figure 8:**
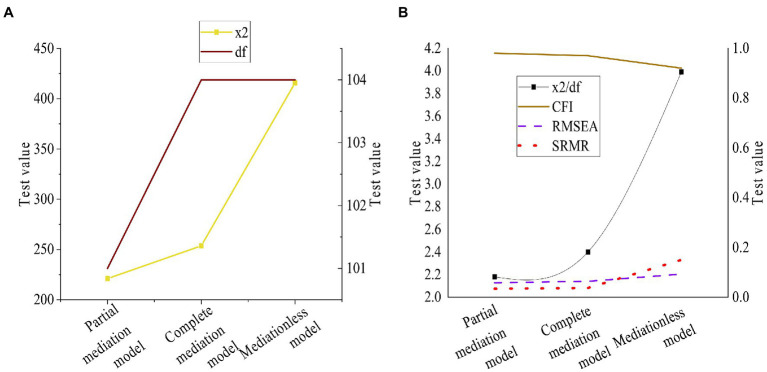
Test results of the structural equation model **(A)**: Chi-square and degrees of freedom; **(B)** Chi-square degrees of freedom, CFI, RMSEA, and NNFI.

Figure 7 describes the p value of the mediating effect of organizational psychological ownership between transformational leaders and employees’ advice to their superiors and peers. In addition, the p value test is conducted on the relation between organizational psychological ownership and employees’ creativity. According to the test results, it is found that the *p*-values of d1, d2, d3, and d4 are less than 0.01. And the standardized root-mean-square residual (SRMR) is introduced, the structural equation model is implemented to verify the reliability of the mediating effect. The models are compared, and the results are shown in Figure 8.

Figure 7 shows that the path coefficients are 0.29, 0.61, 0.53, and 0.59, respectively. These data indicate a mediating effect between the impact of organizational psychological ownership on employees’ creativity in the four quadrants, and hypothesis d1 is verified. The effect of organizational psychological ownership on employees’ creativity in the first quadrant is the largest, so its mediating effect on the first quadrant is the strongest, which verifies hypothesis d2. organizational psychological ownership has a mediating effect on transformational leadership in the influence of the advice to supervisors and colleagues, so d3 and d4 are verified.

In the model test of Figure 8, the chi-square test of the partial mediation model, the complete mediation model, and the mediations model is conducted. The results show that the fitting of the model does not change after transformational leadership and giving advice to supervisors are added to the benchmark model. The fitting of the model does not change after advising colleagues is added to the model. Therefore, the mediating effect tested by the research is reliable, and the hypotheses are verified.

In short, the three hypotheses are as: (1) the changes of employees’ organizational psychological ownership in the four quadrants; (2) the influence of transformational leadership on employees’ advice to their superiors and colleagues; and (3) the role of organizational psychological ownership in transformational leadership, employees’ advice to their supervisors, and colleagues. After these hypotheses are tested, it is found that transformational leadership positively impacts employees’ advice to their supervisors and colleagues. In addition, organizational psychological ownership also has mediating effect in the hypothetical path (*p* < 0.01). Therefore, the intermediary result is reliable, and the hypothesis test is reasonable.

## Conclusion

With the development of science and technology, the market competition becomes more and more fierce. If all kinds of enterprises want to make a breakthrough at the cultural level, employees’ subjective initiative should be totally inspired. First, employees’ perceived HRMS and organizational psychology under the leadership of new enterprises should be classified and defined. Then, the relevant transformational leadership and advice are expounded, and the hypothesis that the four-quadrant types of HRMS have a relevant impact on employees’ creativity is proposed. Through the experiment and modeling test, the path coefficients of employees’ advice to their superiors and colleagues are 0.28 and 0.31, respectively, and the *p* value is less than 0.01 (*p* < 0.01). In the test of the relationship between the four HRMS quadrants and psychological ownership, the r value of the first quadrant is 0.68, the r value of the second quadrant is −0.08, the r value of the third quadrant is −0.11, and the r value of the fourth quadrant is −0.68. This shows that the first quadrant has the greatest impact on employees’ creativity and gradually decreases from the second to the fourth. In the test of mediating effect, the mediating effect of organizational psychological ownership in the hypothetical path (*p* < 0.01) is verified, and the structural equation model of the mediating effect is tested. The results show that organizational psychological ownership has a good mediating effect. Therefore, the research on the impact of employee relations on the innovation behavior of start-ups at the level of organizational psychology and culture has great significance. Due to the limited energy, there are still some limitations. For example, the interpretation angle is relatively single, and the design is not comprehensive. In the follow-up study, enterprises’ owners, leaders, and employees will be introduced to draw richer research conclusions. And longitudinal research will be conducted, and employees’ organizational psychology changes will be discussed.

## Data Availability Statement

The raw data supporting the conclusions of this article will be made available by the authors, without undue reservation.

## Ethics Statement

The studies involving human participants were reviewed and approved by Qinghai University Ethics Committee. The patients/participants provided their written informed consent to participate in this study.

## Author Contributions

All authors listed have made a substantial, direct, and intellectual contribution to the work and approved it for publication.

## Conflict of Interest

The authors declare that the research was conducted in the absence of any commercial or financial relationships that could be construed as a potential conflict of interest.

## Publisher’s Note

All claims expressed in this article are solely those of the authors and do not necessarily represent those of their affiliated organizations, or those of the publisher, the editors and the reviewers. Any product that may be evaluated in this article, or claim that may be made by its manufacturer, is not guaranteed or endorsed by the publisher.
